# The effects of self-management education on self-efficacy, self-esteem, and health behaviors among patients with stroke

**DOI:** 10.1097/MD.0000000000040758

**Published:** 2025-02-14

**Authors:** Runping Li, Dan Zhu, Zhiwen Tan

**Affiliations:** a Department of Encephalopathy, The Central Hospital of Enshi Tujia and Miao Autonomous Prefecture, Enshi, Hubei, China; b Department of Bone Traumatology, The Central Hospital of Enshi Tujia and Miao Autonomous Prefecture, Enshi, Hubei, China.

**Keywords:** health behavior, self-efficacy, self-esteem, self-management education

## Abstract

The concept of self-management has become one of the most critical concepts in stroke rehabilitation. This study examined the impact of a 2-week stroke self-management program on the self-efficacy, self-esteem, and health behaviors of stroke patients. This retrospective study was conducted from January 2022 to October 2022 with 116 patients who had been admitted to the hospital with a stroke diagnosis after admission to the hospital. This study had 4 primary outcomes: self-efficacy, self-esteem, and health behaviors. Each of those outcomes was assessed at baseline, postintervention, and 1 month postintervention. Fifty-one participants were enrolled in the intervention group and 49 participants were enrolled in the control group. According to the baseline data, there was no significant difference between the 2 groups regarding demographics, clinical characteristics, self-efficacy, self-esteem, and health behaviors of participants (*P* > .05). Moreover, the difference between the mean scores of self-efficacy, self-esteem, and health behaviors between the 3 measurement time points was not significant (*P* > .05) in the control group, but there were considerable differences between the mean scores of all 3 factors in the intervention group (*P* < .05). A significant difference was observed between the groups in terms of self-efficacy, self-esteem, and health behaviors after the educational program (*P *< .05). Patients with stroke can significantly improve their self-efficacy, self-esteem, and health behaviors as a result of self-management education.

## 1. Introduction

Stroke is one of the leading causes of long-term disability in the United States, with over 7 million people suffering from stroke at present.^[1]^ Approximately 200 million disability-adjusted life years will be lost every year as a result of stroke by 2030, according to recent projections.^[2]^ There is a high rate of stroke survivors returning home from acute hospitals after at least 45% of them have been hospitalized, usually relying on family members to provide any further support and care.^[3]^ There is still a lack of appropriate and effective educational tools for stroke survivors’ caregivers. This makes it difficult to provide appropriate and effective care when the patient suffers a stroke.^[4]^

Creer et al^[5]^ first described self-management in 1976. They described it as the process of the patient actively participating in their healthcare, based on Bandura’s concept of self-efficacy. It is now widely accepted that self-management can help people manage their daily health care needs associated with chronic diseases by teaching them strategies for managing their daily health care needs.^[6]^ Self-management education improves quality of life, physical functioning, self-efficacy, participation in social activities and activities of daily living, reductions in depression symptoms, and reduced utilization of health care services.^[7,8]^ A reduction in health care utilization has been associated with self-management programs, along with improvements in emotional and physical health, as well as improved quality of life and self-efficacy.^[9]^ Self-esteem (SE) is an attitude toward oneself that fosters a realistic and positive view of oneself as well as a feeling of self-worth. It has been reported that patients suffering from stroke have low SE, and that stroke also negatively affects self-efficacy, which is an intrapersonal factor that influences behavior and disease management.^[10]^

At present, stroke patients’ health behavior status is less than optimistic, as they lack exercise, follow poor eating habits, smoke, drink, and fail to comply with medication instructions.^[11]^ Insufficient health knowledge contributes to patients’ poor health behaviors.^[12]^ Self-management education should, however, be examined in the context of self-efficacy, SE, and health behavior in stroke patients.

In the current article, the authors present the results of a randomized controlled trial that attempted to determine whether self-management education was effective in improving health outcomes for individuals with stroke.

## 2. Methods

### 2.1. Study design and participants

This retrospective study was approved by the Ethics Committee of Enshi Ethnic Hospital (No. 2022-016-K014). This study was a single-center study. A total of 116 individuals were randomly assigned to either the intervention group or the control group while inpatients in the neurology department of a hospital, from January 2022 to October 2022.

According to the criteria for inclusion, the following criteria were met: diagnosis of stroke including hemorrhagic stroke, ischemic stroke, and subdural hemorrhage; a minimum age of 18 years; capable of understanding the content of the questionnaire as well as writing and communicating verbally; willing to participate in the follow-up study after 1 month.

These criteria were used to exclude participants: an established history of serious liver disease, kidney disease, or malignant tumor; psychiatric symptoms or mental illness; involvement in any other health education research after discharge.

### 2.2. Intervention

Randomly selected participants were divided into 2 groups: the control groups received routine health education and the intervention groups received self-management education.

### 2.3. A routine health education program

Nursing staff provided health education, stroke prevention, and treatment to the control group, once every 2 days during hospitalization.

### 2.4. A program for self-management education

Based on the conceptual framework proposed by Strauss and Corbin, the study intervention focused on education and support for self-management. The researchers conducted 30-minute sessions every 2 days as part of this program. Table 1 summarizes the self-education program.

**Table 1:** Summary of the self-education program.

An overview of the self-management education	Strategic management
1. Knowledge about health care	Discussions about problem-solving and education
2. Taking steps to deal with illness in advance	Brainstorming and educational discussions
3. Capacity for self-care	Discussions and educational sessions in groups
4. Achieving coping skills management	Problem-solving and participation in groups
5. Spirituality	Discussions in groups and brainstorming
6. Managing emotions	Brainstorming and solving problems
7. Conditions of social management	Discussions and problem-solving for educational purposes

### 2.5. Follow-up telephone calls following discharge

A telephone call was made to the patient 1 month after discharge. A half-hour was spent on telephone follow-ups.

At 3 different time points, namely before, immediately after, and 1 month after the intervention, both groups completed the study instruments.

## 3. Data collection methods

### 3.1. Demographic and disease-related data

Data regarding demographics and diseases were collected using a self-designed questionnaire. A variety of demographic data was collected, including gender, body mass index (BMI), age, education level, stroke classification, smoking history, coronary heart disease, and diabetes history. Besides, Scherer’s self-efficacy (SEF) Scale,^[13]^ Rosenberg SE scale,^[14]^ and the Health Behavior Scale for Stroke Patients (HBS-SP)^[15]^ were also used in this study.

### 3.2. Scherer’s SEF Scale

Scherer’s SEF Scale measures individuals’ beliefs about their ability to cope with various situations through 17 items on the general SEF. Each item is evaluated on a 5-point scale from 1 to 5, with a total possible score of 17 to 85.^[13]^ The SEF scale has been found to be reliable with a Cronbach’s α of 0.84 in a previous study.^[16]^

### 3.3. Rosenberg SE Scale

The Rosenberg SE Scale is a widely used SE assessment tool. It contains 5 positively framed items (i.e., items 1–5), as well as 5 items that are negatively framed (i.e., items 6–10). Each item on a 0 to 3 scale can be 0 to 30. Scores greater than 25 indicate a high SE, scores between 15 and 25 indicate a moderate SE, and scores less than 15 indicate a low SE. In previous studies, Cronbach’s α was 0.74, indicating the validity and reliability of this scale.^[17]^

### 3.4. Health Behavior Scale for Stroke Patients

The Health Behavior Scale for Stroke Patients was developed by Zhang et al.^[15]^ The scale consists of 25 items and 7 dimensions, including exercise, medication compliance, nutrition, a low-sodium and low-fat diet, and monitoring blood pressure. Smoking cessation, alcohol restriction, and food restriction are also included on the scale. This scale examines stroke patients’ health behaviors during the past month. Based on a Likert grade 4 scoring method, the scale allocates 1 to 4 points to each of the following: “never,” “sometimes,” “often,” and “always.” In general, the higher the score, the healthier the patient’s behavior is. Previously, Cronbach’s α was 0.88.

### 3.5. Procedures

A study coordinator identified potential enrollment patients after each patient was admitted to the hospital. The identifiers of groups were sequentially sequenced at random. The identification numbers for the groups were printed on folded papers and sealed in the same opaque envelope by persons who were not involved in the research. Study coordinators assigned serial numbers to each envelope. Afterward, participants were randomly assigned to either the control group (n = 58) or the intervention group (n = 58).

Informed consent has been obtained from all patients and baseline data has been collected. The questionnaire was explained to each patient before completion. In order to provide stroke education, the questionnaires were completed within 24 hours of admission. Results were measured by investigators blinded to patient allocation. Data were entered by a researcher without a connection to the patient.

### 3.6. Ethical

In accordance with the Helsinki Declaration of 1975, the study was conducted according to ethical principles. An ethics committee at the hospital where the study was conducted approved the study. Before participating in the study, all patients signed an informed consent form.

### 3.7. Data analysis

Data processing was carried out using SPSS 20.0 software. The significance level for the study was set at 0.05. Individual sample *t* tests or Student *t* tests were applied to compare the differences between the 2 groups. To compare the scores of patients before and after intervention and 1 month after intervention, a multivariate repeated measure analysis of variance (ANOVA) was used.

## 4. Results

### 4.1. Participant recruitment and retention

**Figure 1.**
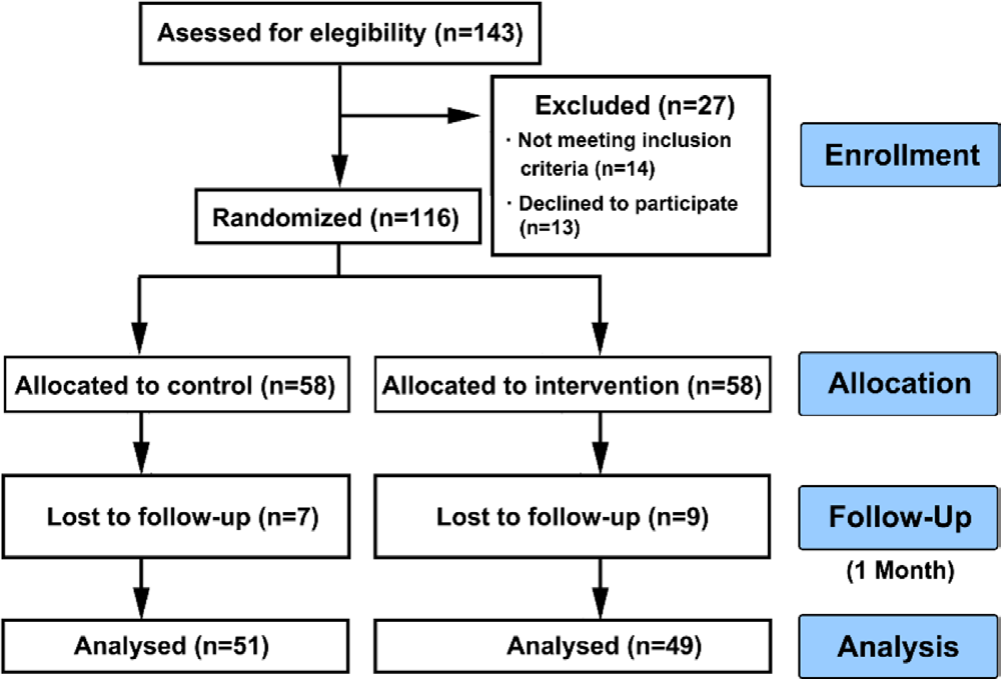
The flow diagram of participant’s enrollments.

The study recruited 143 patients, and 116 were enrolled in the study. The patients were divided equally between the intervention and control groups, with 58 in each group. Forty-nine patients in the intervention group (84.48%) and 51 patients in the control group (84.48%) completed the 1-month follow-up survey. Loss of contact was the primary cause of attrition during the study. Figure 1 illustrates participants’ enrollments.

### 4.2. Demographic and disease-related data

In the comparison of baseline characteristics and disease-related data between the control group (n = 51) and the intervention group (n = 49), no significant differences were observed. In terms of gender distribution, there were 29 males and 22 females in the control group, and 29 males and 20 females in the intervention group, with a *P* value of .19, indicating no significant difference in gender distribution between the 2 groups. For BMI, the average BMI was 22.67 ± 2.27 in the control group and 23.11 ± 2.88 in the intervention group, with a *P* value of .78, suggesting no significant difference in BMI between the groups. The mean age in the control group was 63.15 ± 5.35 years, while it was 62.26 ± 5.45 years in the intervention group, with a *P* value of .20, showing no significant age difference. Regarding educational level, 8 participants in the control group and 6 in the intervention group had a university degree; 10 in the control group and 12 in the intervention group had a diploma; 15 in the control group and 17 in the intervention group had education below a diploma level; and 18 in the control group and 14 in the intervention group were illiterate. The *P* value of .87 indicates no significant difference in educational levels between the 2 groups. For stroke classification, there were 22 patients with cerebral thrombosis in the control group and 18 in the intervention group; 20 with cerebral embolism in the control group and 23 in the intervention group; and 9 with lacunar cerebral infarction in the control group and 8 in the intervention group, with a *P* value of .72, showing no significant difference in stroke classification. Regarding smoking history, 32 patients in the control group had a smoking history, while 19 did not; in the intervention group, 39 had a smoking history, and 9 did not, with no significant difference (*P* = .18). For coronary heart disease, 21 patients in the control group and 18 in the intervention group had a history of the condition, with a *P* value of .55, indicating no significant difference between the 2 groups. Regarding diabetes history, there were 27 patients in the control group and 24 in the intervention group, with a *P* value of .57, showing no significant difference. In summary, the above results demonstrate that there were no significant differences in baseline characteristics between the control and intervention groups (Table 2).

**Table 2:** PANSS sub-score assessment of schizophrenia severity.

Group	Positive symptoms		Negative symptoms		General psychopathology symptoms	
	Before care	After care	Before care	After care	Before care	After care
n	72	72	72	72	72	72
Control group	29.16 ± 2.46	27.41 ± 2.12*	10.12 ± 1.21	7.46 ± 1.96*	45.63 ± 3.26	27.64 ± 4.16*
Experimental group	30.04 ± 2.33	25.23 ± 2.22*	10.45 ± 1.03	6.62 ± 1.47*	46.12 ± 2.68	22.56 ± 3.11*
*T* value	−1.12	2.20	−0.02	1.36	−0.13	4.26
*P* value	.45	.01	.51	.02	.44	<.001

* Significant difference between the 2 groups (*P* < .05).

### 4.3. Findings related to self-efficacy and SE

**Figure 2.**
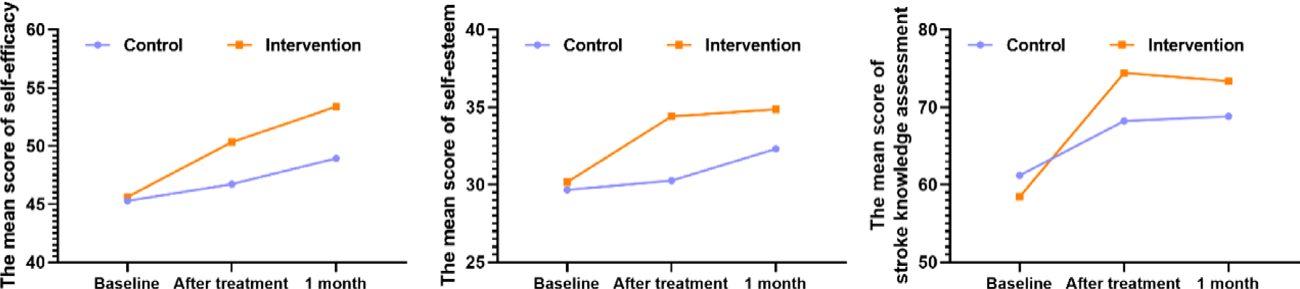
Comparison of patients’ self-efficacy and self-esteem in intervention and control groups.

Although the repeated measures ANOVA results revealed significant differences between self-efficacy and SE in the intervention group during the 3 measurement stages (baseline, postintervention, and 1 month postintervention) (*P* < .05). Compared with the control group, the self-efficacy and SE of the intervention group significantly improved in the postintervention and 1 month following the intervention (Table 3 and Fig. 2).

**Table 3:** Comparison of patients’ self-efficacy and self-esteem in intervention and control groups.

Outcomes	Control (n = 51)	Intervention (n = 49)	*P* value
Self-efficacy			
Baseline	45.29 ± 9.36	45.63 ± 9.68	.08
Postintervention	46.72 ± 6.90	50.34 ± 9.63	.03
1 mo post-intervention	48.94 ± 7.67	53.39 ± 10.46*	.02
* P* value	.78	<.001	
Self-esteem			
Baseline	29.67 ± 4.82	30.17 ± 7.74	.70
Postintervention	30.29 ± 4.87	34.42 ± 8.37†	.005
1 mo postintervention	32.32 ± 6.09	34.87 ± 6.49‡	.04
* P* value	.05	.003	

### 4.4. Findings related to HBS-SP

**Figure 3.**
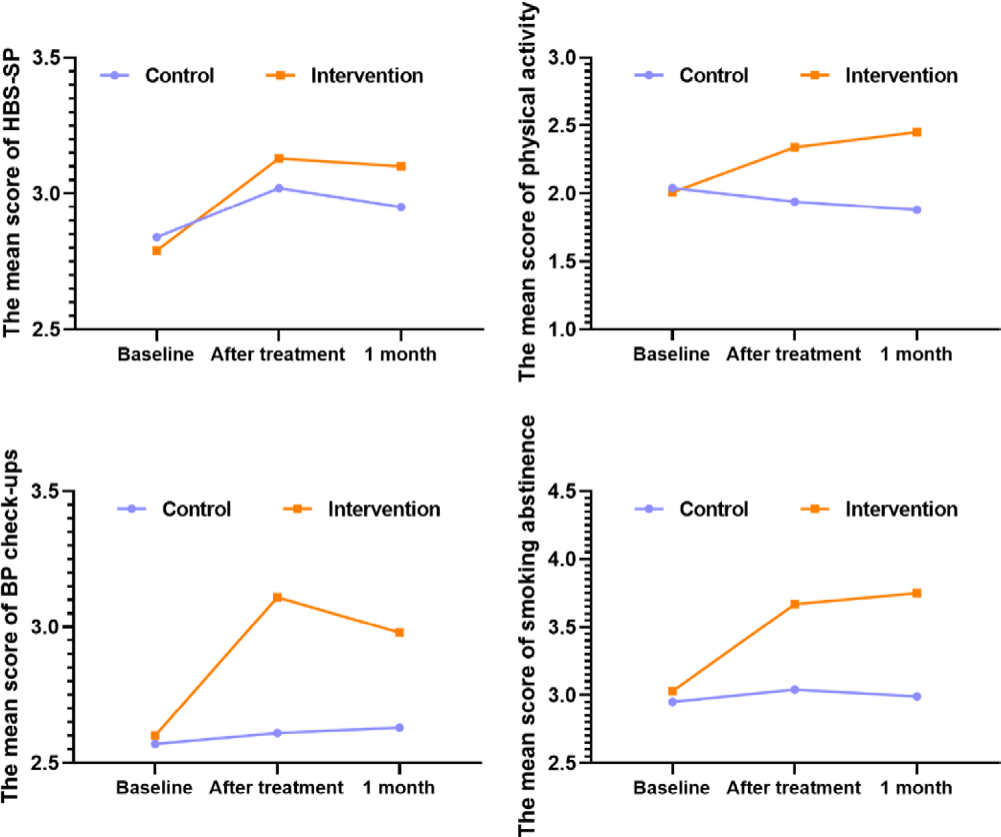
The difference of HBS-SP between the 2 groups at 3 time points. HBS-SP = Health Behavior Scale for Stroke Patients.

A repeated measures ANOVA revealed that variations in the total score across 3 measurement time points of HBS-SP, physical activity, blood pressure checkups, smoking abstinence, and medication adherence were significant in the intervention group (*P* < .05) and insignificant in the control group (*P* > .05). A comparison between the intervention and control groups revealed significant differences regarding the variations in HBS, physical activity, blood pressure checks, and smoking abstinence (*P* < .05) (Table 4 and Fig. 3).

**Table 4:** The difference of HBS-SP between the 2 groups at 3 time points (mean ± SD).

Outcomes	Control (n = 51)	Intervention (n = 49)	*P* value
Health Behavior Scale (total score)			
Baseline	2.84 ± 0.57	2.79 ± 0.61	.63
Postintervention	3.02 ± 0.69	3.13 ± 0.73*	.01
1 mo postintervention	2.96 ± 0.62	3.10 ± 0.59†	.004
* P* value	.24	.01	
Physical activity			
Baseline	2.04 ± 0.59	2.01 ± 0.68	.57
After treatment	1.94 ± 0.57	2.34 ± 0.76	.004
1 mo postintervention	1.88 ± 0.73	2.45 ± 0.84†	.001
* P* value	.52	.01	
Nutrition			
Baseline	2.16 ± 0.98	2.09 ± 0.94	.66
After treatment	2.34 ± 0.86	2.28 ± 0.48	.66
1 mo postintervention	2.29 ± 0.75	2.34 ± 0.82	.75
* P* value	.47	.24	
Low-salt diet			
Baseline	2.49 ± 0.93	2.55 ± 0.88	.74
After treatment	2.44 ± 0.86	2.36 ± 0.79	.62
1 mo postintervention	2.53 ± 1.04	2.62 ± 0.58	.59
* P* value	.89	.21	
BP checkups			
Baseline	2.57 ± 1.02	2.60 ± 0.83	.87
After treatment	2.61 ± 0.97	3.11 ± 0.65‡	.003
1 mo post-intervention	2.63 ± 0.85	2.98 ± 0.83†	.03
* P* value	.94	.004	
Smoking abstinence			
Baseline	2.95 ± 0.96	3.03 ± 0.12	.56
After treatment	3.04 ± 1.14	3.67 ± 0.86‡	.002
1 mo postintervention	2.99 ± 1.04	3.75 ± 1.39§	.002
* P* value	.91	<.001	
Limiting alcohol use			
Baseline	3.01 ± 1.05	2.98 ± 0.84	.87
After treatment	3.02 ± 0.91	3.07 ± 1.05	.79
1 mo postintervention	3.06 ± 0.79	3.04 ± 0.60	.88
* P* value	.95	.86	
Medication adherence			
Baseline	3.36 ± 1.20	3.32 ± 0.99	.74
After treatment	3.53 ± 1.48	3.74 ± 0.66	.66
1 mo postintervention	3.53 ± 1.16	3.69 ± 0.80	.08
* P* value	.52	.02	

HBS-SP = Health Behavior Scale for Stroke Patients.

## 5. Discussion

A significant improvement was observed in self-efficacy, SE, and health behaviors after self-management education was provided to stroke patients after intervention and after 1 month following intervention. During the 2 measurement stages, the improvements in health behaviors in the intervention group showed a significantly higher trend than in the control group in terms of total health behaviors and 3 dimensions—physical activity, blood pressure checks, and smoking abstinence.

Similarly to the present study, the results of a study by Lo et al^[18]^ concerning the impact of a series of multimodal self-management behaviors on self-efficacy and outcome expectations improvement in stroke patients were concordant. Despite the fact that they concluded that multimodal self-management strategies could improve patients’ self-efficacy, the variety of the interventions led to a 60% rate of attrition of participants.

An objective of the present study was to improve the self-efficacy of stroke patients, which was consistent with previous studies that examined the effects of self-care education on the self-efficacy of hospitalized patients with myocardial infarction who experienced sudden attacks resulting from vascular disorders. According to the Chronic Disease Self-Efficacy Scales, self-care education increased self-efficacy.^[19]^ During the predischarge planning process, a patient-centered self-management empowerment intervention was shown to be effective for stroke survivors according to a longitudinal study.^[20]^ A significant improvement in self-efficacy was observed after the intervention at 1 month. It has been reported that Lee et al^[21]^ examined participation-based stroke self-management interventions in a day rehabilitation setting in a quasi-experimental study. The researchers found that self-efficacy scores increased in both groups before and after the intervention. In spite of the fact that a significant difference was not observed between the experimental and control groups, this trend of increased self-efficacy was higher in the control group.^[21]^

The SE factor serves as an intermediary (mediating) variable to explain the correlation between increased identity discrepancy and lower mood and poorer quality of life. SE could be impacted by discrepancy when a person is unable to reach goals related to his or her pre-injury identity as a result of reduced self-efficacy.^[22]^ In turn, SE may influence outcomes by reducing the impact of stressful events in a person’s life and facilitating adjustment to illness, psychosocial functioning, and quality of life.^[23]^ When individuals have low SE, they are less likely to be motivated for tasks and less likely to persevere, even when confronted with challenging, anxiety-provoking demands; as a result, they may avoid challenges altogether or quit before they start. This could lead to a reduction in social participation, a decrease in quality of life, and a decrease in opportunities for rewarding engagement. This in turn could result in depression. Educating and supporting patients with epilepsy about self-management significantly improves their self-efficacy, SE, and quality of life.^[16]^

This study showed significantly higher scores 1 month after intervention for health behavior as an outcome, and for some dimensions of the scale as well. As a result of these findings, self-management education interventions improve behaviors to some extent. The total score of health behavior of patients 1 month after the intervention was statistically higher than that of the control group. This indicates that the self-management education intervention was more effective than the control group at enhancing health behavior. Self-management education interventions may significantly reinforce health beliefs to enhance health behaviors.

There may be another reason for the improved health behavior of participants in the self-management education program to increase their utilization of chronic disease resources in stroke patients. According to a report from the American Heart Association, stroke patients’ health behaviors improve if chronic disease resources are better utilized.^[24]^ Using monthly online follow-ups by physicians, a randomized controlled trial of stroke patient management in rural China showed health behavior and physical activity effects.^[25]^ A study conducted in this study found that patients in the intervention group were more likely to utilize chronic disease resources than those in the control group. The self-management program was more convenient, comprehensive, and professional for intervention patients. In addition, this study indicated that the physical activity, medication compliance, smoking abstinence, and blood pressure monitoring scores in the intervention group were significantly higher than the baseline scores at 0 and 1 month after intervention. Interventions may contribute to this.

This study still has some limitations. First, the data in this study mainly relied on self-reported questionnaires, which can lead to subjective bias. Because patients may be affected by memory bias or social expectation effects, the accuracy of the data may be limited, especially when assessing self-efficacy, SE, and health behaviors. In addition, the study only had 1 month of follow-up, so it could not assess the long-term effects of self-management education. Longer follow-up may reveal the persistent impact of interventions on patients’ long-term health behaviors, recurrence rates, and improved quality of life. Future studies should consider multicenter data to improve the external validity of the results. Finally, although this study balanced baseline characteristics between groups, it could not exclude other potential confounding factors (such as socioeconomic status, family support, etc.) for the effect of the intervention.

As a result of the self-report nature of the questionnaires utilized in this study, there may be some bias resulting from self-reporting. Besides, only 1-month follow-up was conducted on the patients, and the long-term effects of self-management were not examined.

## 6. Conclusion

In stroke patients who received self-management education 1 month after intervention, it was found that it improved their self-efficacy, SE, and health behaviors.

## Author contributions

**Conceptualization:** Runping Li, Dan Zhu, Zhiwen Tan.

**Data curation:** Runping Li, Dan Zhu, Zhiwen Tan.

**Formal analysis:** Runping Li, Dan Zhu, Zhiwen Tan.

**Investigation:** Runping Li, Dan Zhu, Zhiwen Tan.

**Validation:** Runping Li.

**Writing—original draft:** Runping Li, Dan Zhu, Zhiwen Tan.

**Writing—review & editing:** Runping Li, Dan Zhu, Zhiwen Tan.

**Methodology:** Dan Zhu, Zhiwen Tan.

**Supervision:** Dan Zhu, Zhiwen Tan.
